# Histopathology of non-mass-like breast lesions on ultrasound

**DOI:** 10.1007/s10396-023-01286-y

**Published:** 2023-02-11

**Authors:** Rin Yamaguchi, Hidetaka Watanabe, Yutaro Mihara, Miki Yamaguchi, Maki Tanaka

**Affiliations:** 1 Department of Pathology and Laboratory Medicine, Kurume University Medical Center, 155-1 Kokubu, Kurume, Fukuoka 839-0863 Japan; 2 Department of Surgery, Japan Community Healthcare Organization Kurume General Hospital, Kurume, Fukuoka Japan; 3 Department of Pathology, Kurume University School of Medicine, Kurume, Fukuoka Japan

**Keywords:** Breast, Carcinoma in situ, Ultrasound, Non-mass-like lesion, Calcification

## Abstract

There have been several investigations of non-mass-like (NML) lesions on ultrasound (US) since Uematsu first described this approach, and it is a relatively new concept for breast examination. However, the results have varied, and there have been only a few studies related to the detailed histopathology of NML lesions on US. Here, we review the histopathology of NML lesions. NML lesions are pathologically benign, atypical, or malignant. There are two major findings of NML lesions on US: architectural distortion and calcifications. Architectural distortion pathologically indicates a fibrous change with ductal proliferation, invasive breast carcinoma, and carcinoma in situ. Histopathologically, microcalcifications are seen in both benign and malignant lesions, and it is important to distinguish between these lesions among NML lesions, particularly fibrocystic changes including adenosis and hyperplasia in the case of benign lesions and carcinoma in situ (ductal and lobular) in the case of malignant lesions. The differential major points may be whether NML lesions are associated with abundant hyperechoic foci, which indicate comedo necrosis on histology. They are usually high-grade carcinoma in situ that may be positive for HER2 or triple negativity. A recent report indicated that low-grade carcinoma in situ showed better survival than higher-grade carcinoma in situ, which is often accompanied by comedo necrosis on histology, reflecting visible microcalcification on US. NML lesions are considered to include a certain rate of low-grade carcinoma in situ. Therefore, more caution may be needed when detecting and managing NML lesions to avoid overdiagnosis and overtreatment as a result of this recent “low-risk ductal carcinoma in situ” concept.

## Introduction

Detection of non-mass-like (NML) lesions of the breast on ultrasound (US) is a relatively new concept in breast examination since it was introduced by Uematsu [1]. The Breast Imaging Reporting and Data System (BI-RADS) lexicon for US provides a definition of breast masses, but hypoechoic areas lack a conspicuous margin or shape and do not meet these criteria; that is, NML lesions. High-resolution breast US often reveals NML lesions in daily practice, and NML lesions in terms of US findings of the breast can be classified as ductal or non-ductal. Among them, ductal carcinoma in situ (DCIS) is often identified as an NML lesion on US. Furthermore, NML lesions have not only been found to be DCIS but also other malignancies and benign lesions. Therefore, identification of NML lesions and their differentiation are important issues. Although the concept of NML lesions is becoming mainstream, there are only a few detailed reports of them from the perspective of pathological findings and their potential malignancy. Here, we have reviewed NML lesions and their pathological findings, including breast cancer subtypes, and discuss future directions.

### Associated findings of NML lesions related to histology

**Fig. 1:**
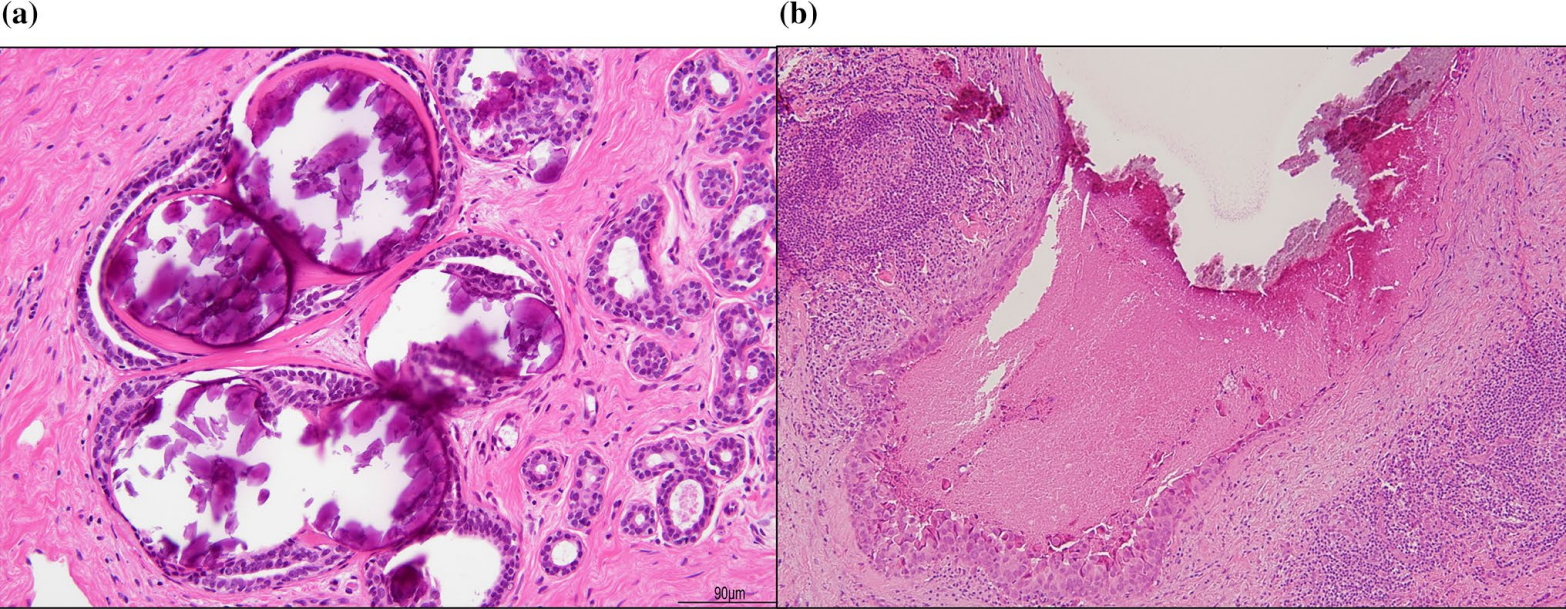
Pathological calcification. **a** Secretory-type (psammoma body-like calcification) calcification in blunt duct adenosis with a benign background. **b** Necrotic-type, which is usually seen in the background with comedo necrosis

Uematsu [1] noted two important categories of the associated findings of NML lesions on US, namely architectural distortion and calcifications. Architectural distortion pathologically indicates a biopsy scar, fibrosis following neoadjuvant chemotherapy, sclerosing adenosis, radial scar, invasive carcinoma, and DCIS. Calcifications are not always definitively identified on US, but they can be recognized as echogenic foci in a hypoechoic area [1]. They are considered to pathologically indicate necrosis and/or subsequent calcification of debris in malignant in situ lesions, including DCIS and lobular carcinoma in situ (LCIS) in general, like calcifications detected on mammography. Histopathologically, microcalcifications occur in both benign and malignant lesions. Tabor and Dean [2] noted calcifications that were related to the mammographic appearance. Calcifications that were not malignant or of epithelial origin were classified as miscellaneous, and malignant-type calcifications within ducts and/or in terminal ductal lobular units were classified into four groups: casting type, skipping stone-like, crushed stone-like/pleomorphic, and powdery/cotton ball-like. Psammoma body-like calcifications include both benign and malignant lesions. In Japan, pathological calcifications are divided into three types: secretory (including psammoma body-like calcifications), according to the epithelial origin, which are benign lesions and usually grade 1 carcinoma in situ (Fig. 1a); necrotic, which is considered to be caused by comedo necrosis or cellular debris in general (Fig. 1b); and stromal, which is caused by stromal hyalinization [3]. They can all be detected by US. Additionally, recent advances in US technology may allow the detection of conducted secretion.

### Frequencies of benign, atypical, and malignant NML lesions

There have been several reports of pathologically confirmed NML lesions. Lee et al. [4] reported 29 benign (90.6%), one atypical (3.1%), and two (6.3%) malignant lesions among 32 lesions. Wang et al. [5] examined 80 NMLs and found that 37 were benign (46.2%) and 43 were malignant (53.8%). Park et al. [6] reported that 88 (72.7%) were benign and 33 (27.3%) were malignant. Park et al. [7] examined a larger number of 715 NMLs and found that 385 (53.8%) were benign and 330 (46.2%) were malignant. Kim et al. [8] found that 97 (69.7%) were benign and 42 (30.2%) were malignant among 139 NMLs. Among 42 malignancies first detected as NML lesions, six cases had concomitant mass-forming cancers in this study. Overall, it appears that the detection frequency of benign, atypical, and malignant lesions varies depending on the institution. As the concept of NML lesions becomes more widespread, the frequencies of benign and malignant NML lesions may change.

### Histopathology of benign and atypical NML lesions

There are several reports of histological types of benign NML lesions. Lee et al. [4] reported 29 benign lesions and one atypical lesion assessed by needle biopsy among 32 lesions. The benign lesions had fibrocystic changes, adenosis, ductal hyperplasia, inflammation, pseudoangiomatous stromal hyperplasia, and intraductal papilloma, and the atypical lesion was atypical ductal hyperplasia. Wang et al. [5] examined 80 NML lesions and found 37 benign (46.2%), 31 adenosis, four papilloma, and two inflammatory lesions. Park et al. [6] identified 82 benign lesions among 115 NML lesions, of which 46 had fibrocystic changes, adenosis, fibroadenomatoid hyperplasia, and fibrosis (56.1%); six had mastitis, abscess, and duct ectasia (7.3%); one had diabetic mastopathy; two were papilloma; and one was atypical ductal hyperplasia. Park et al. [7] examined two datasets of 460 and 255 cases, and the combined data of benign lesions (247 and 138, respectively) revealed that 67 had fibrocystic changes, 67 had fibrosis, 20 had adenosis, 17 were fibroadenoma, 15 had a columnar cell change, 13 were papilloma, and 28 were other benign lesions. Although the terminologies of histological evaluation differ among institutions, benign NML lesions exhibit mainly fibrocystic changes and adenosis and relatively fewer inflammatory changes. Additionally, there is the possibility of benign tumors, such as papilloma and fibroadenoma, in these studies.

### Histopathology of malignant NML lesions

It has been reported that the histological types of malignant NML lesions are invasive (infiltrating) ductal carcinoma, DCIS including papillary DCIS, invasive lobular carcinoma, microinvasive or minimally invasive carcinoma, a mixed type of lobular and ductal including LCIS and DCIS, LCIS, and mucinous or other invasive cancers [1, 4–8]. Although NML lesions in some studies have mainly been invasive carcinoma, it is important to distinguish between carcinoma in situ (DCIS and LCIS) and benign NML lesions. Additionally, DCIS in sclerosing lesions, including adenosis, radial scar, and complex sclerosing lesions, may be detected as NML lesions on US. Although it has been noted that the DCIS range of NML lesions was 11%–42%, Watanabe et al. reported that approximately 60% of DCISs were NML lesions in their study [9].

### Relationship between calcifications or necrosis in US findings and carcinoma in situ of NML lesions

**Fig. 2:**
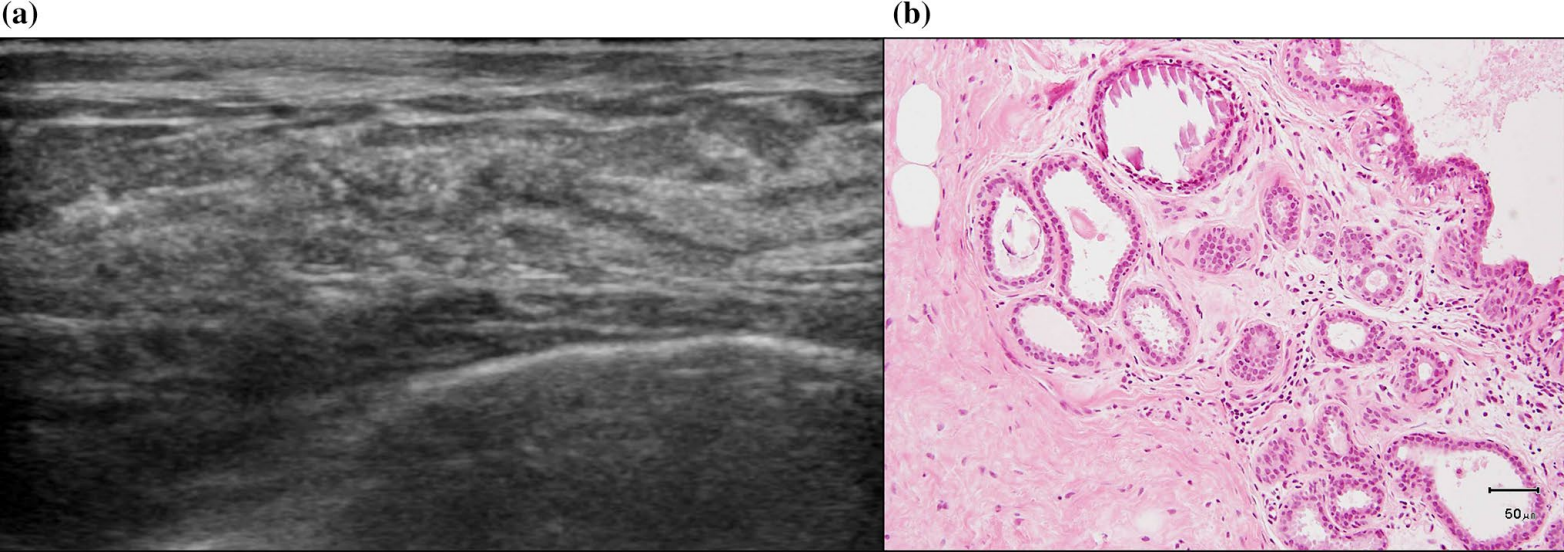
**a** Non-mass-like lesions on ultrasound with some unclear echogenic foci. **b** Histology was blunt duct adenosis with secretion and secretory-type calcification in the ducts

**Fig. 3:**
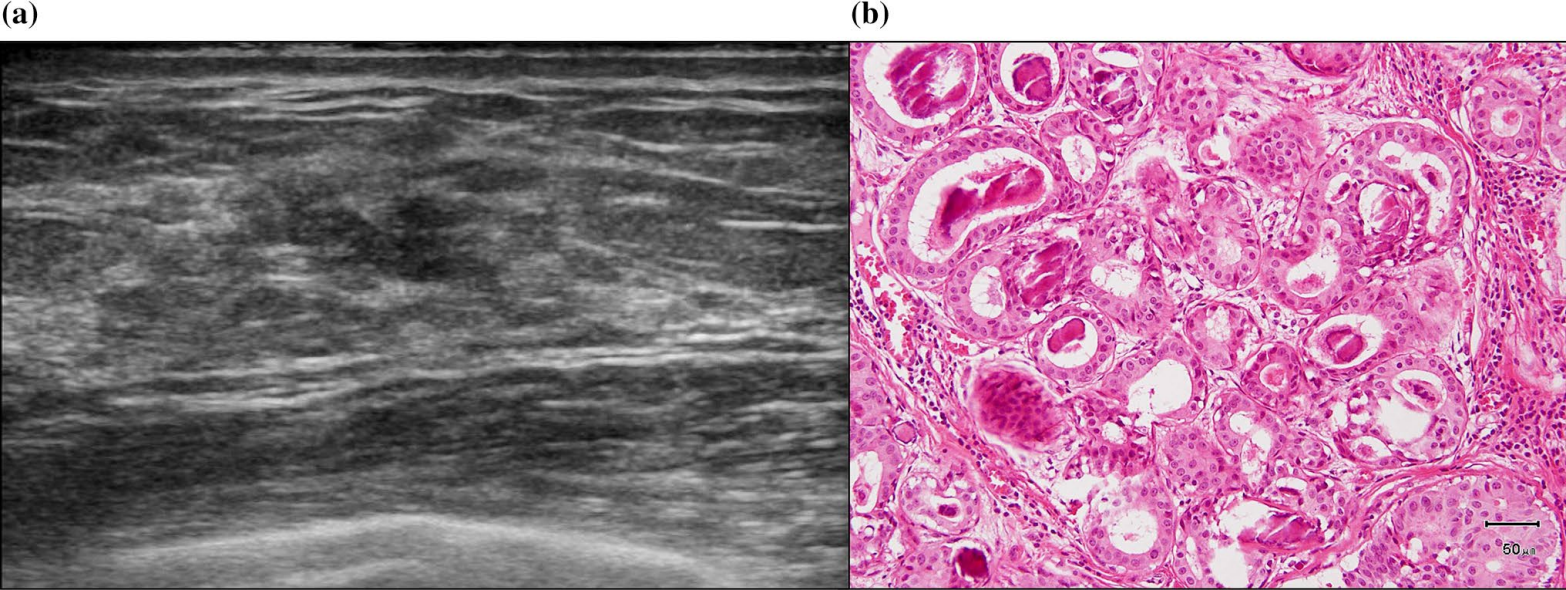
**a** Non-mass-like lesions on ultrasound with some unclear echogenic foci. **b** Histology was low-grade ductal carcinoma in situ with secretory-type calcification

**Fig. 4:**
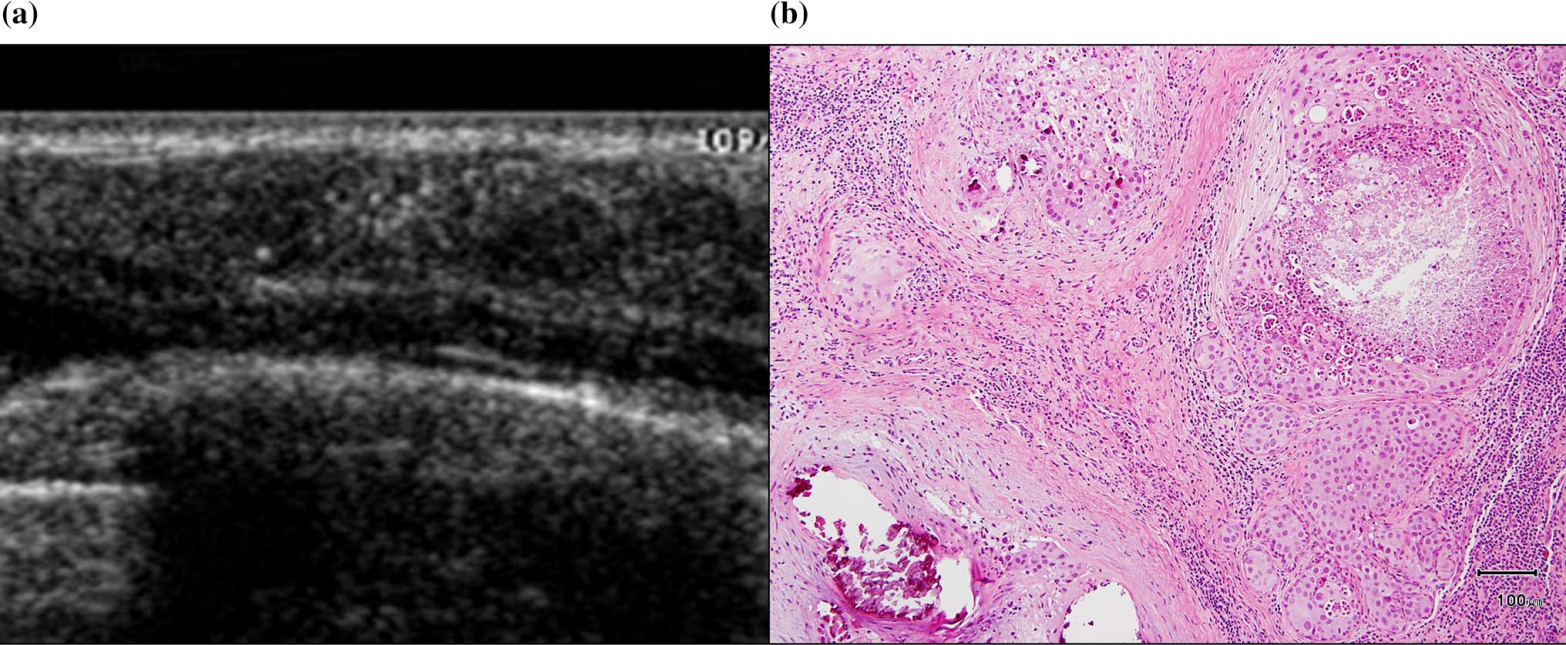
**a** Non-mass-like lesions on ultrasound with prominent hyperechoic foci. **b** Histology was high-grade ductal carcinoma in situ with comedo necrosis and necrotic-type calcification with healing in the ducts

Calcifications on US indicate benign and malignant lesions [6]. Benign lesions, such as blunt duct adenoses with secretory-type calcifications, are indicated by echogenic foci in NML lesions (Fig. 2a and b). Carcinoma in situ is DCIS including the papillary type and LCIS. Low-grade (LG) DCIS with secretory-type calcification is also indicated by NML lesions with unclear echogenic foci, similar to benign lesions (Fig. 3a and b). Uematsu [1] found that some NML lesions were DCIS, of which one case was LG-DCIS with multiple irregular distended ducts containing cast-like echogenic components with many penetrating vessels on a color Doppler sonogram. High-grade (HG) DCIS showed clustered punctate echogenic foci without a mass on US (Fig. 4a and b). Jin et al. [10] reported that seven cases with microcalcifications were detected by US among 15 NML lesions (indistinct hypoechogenicity) out of 129 DCISs. Wang et al. [5] found that hypoechogenic areas with sporadic or clustered microcalcifications were significantly associated with malignant lesions and showed a high positive predictive value (78.3%) for carcinoma. Watanabe et al. [9] found comedo necrosis and echogenic foci at a rate of 26% in 50.9% of NML lesions on US among 705 DCIS lesions in the Japan Association of Breast and Thyroid Sonology (JABTS) multicenter, retrospective observational study (JABTS BC-02 study). These echogenic foci may indicate comedo necrosis and/or calcifications.

### Relationships between low- and high-grade pathways and subtypes of carcinoma in situ

Recently, carcinoma in situ was divided into two pathways: LG and HG [11, 12]. LG pathways are usually low risk and HG pathways are relatively high risk. LG carcinoma in situ has a mild–moderate nuclear grade and is typically without or with a relatively small amount of comedo necrosis. However, HG carcinoma in situ has a high nuclear grade and usually abundant comedo necrosis [11, 12]. Interestingly, Gunawardena and Taylor [13] investigated the risk of NML lesions versus mass-like lesions on US patterns in DCIS and concluded that NML lesion morphology on US was associated with the histological features of high-risk DCIS with higher rates of necrosis than mass-like lesions.

**Fig. 5:**
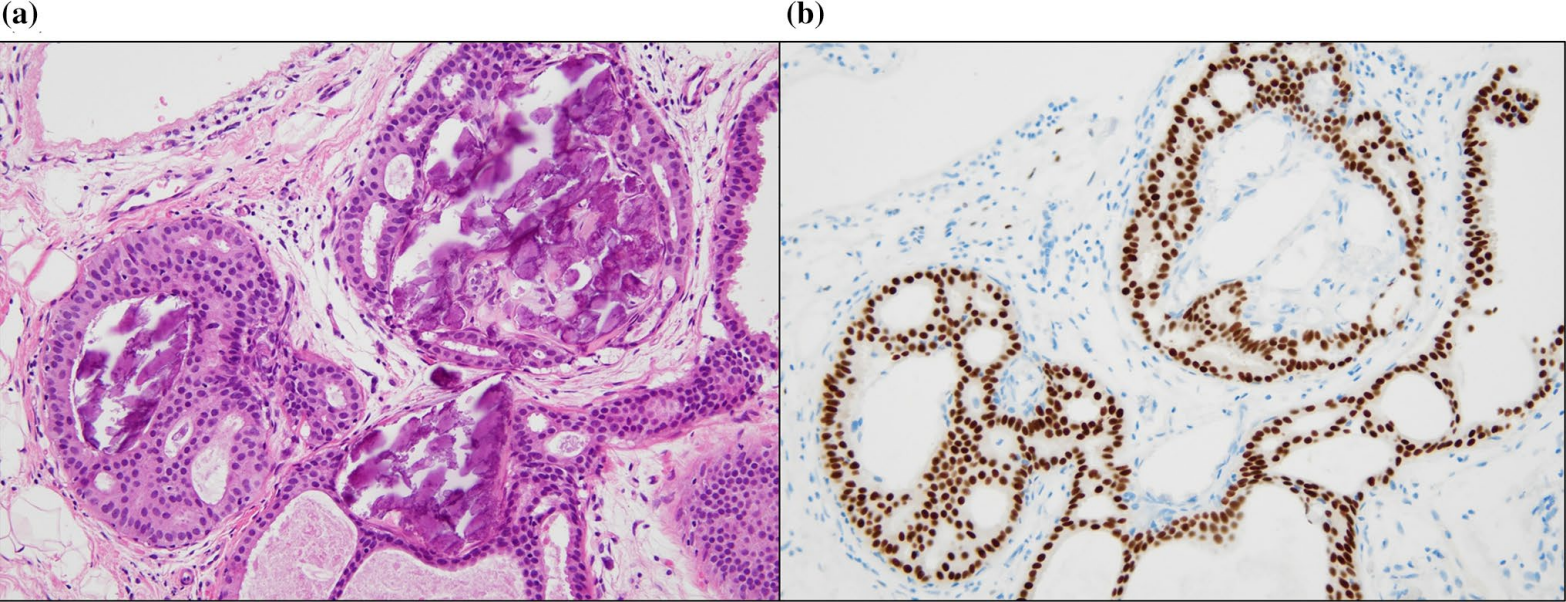
**a** Low-grade carcinoma in situ with secretory-type calcifications. **b** Low-grade carcinoma in situ with secretory-type calcifications is related to estrogen receptor expression

**Fig. 6:**
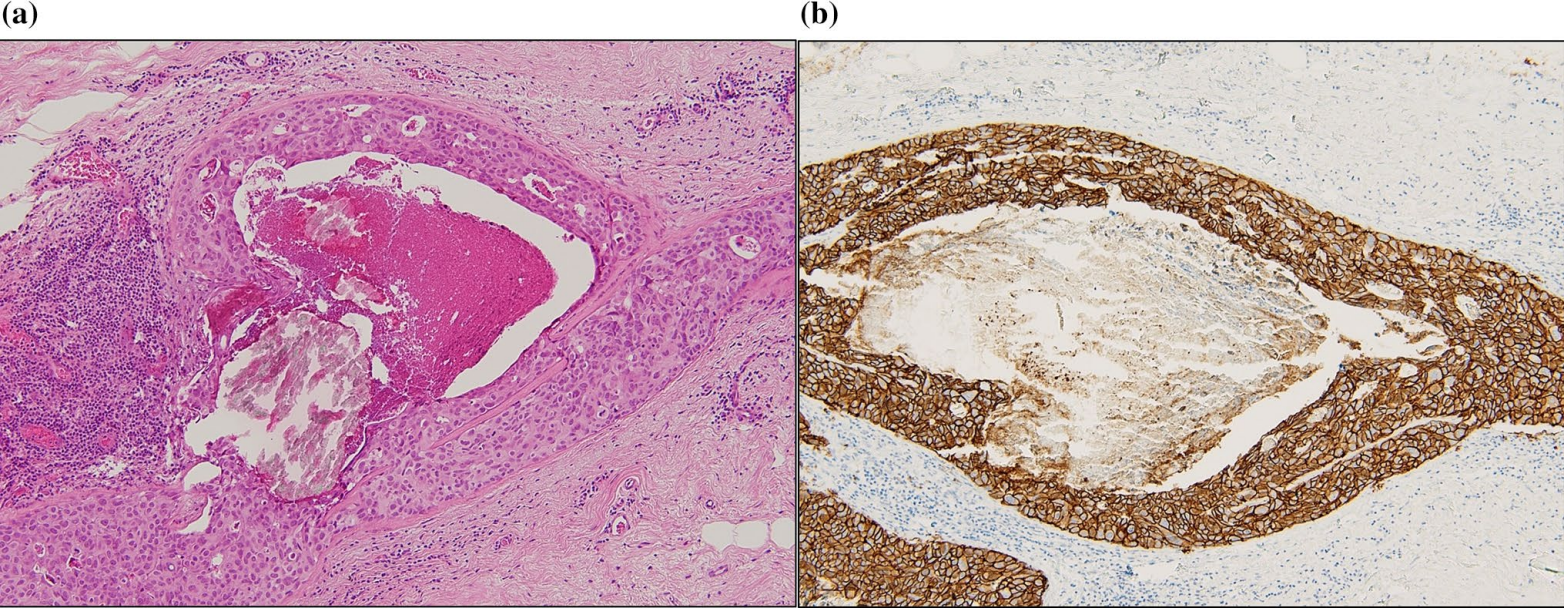
**a** High-grade carcinoma in situ with abundant comedo necrosis and necrotic-type calcification. **b** High-grade carcinoma in situ with abundant comedo necrosis and necrotic-type calcification is related to HER2 expression

LG carcinoma in situ with secretory-type calcifications is usually related to estrogen receptor expression (Fig. 5a, b), while HG carcinoma in situ (mainly DCIS) with abundant comedo necrosis is typically related to HER2 expression (Fig. 6a, b) [11, 12]. The relationships of NML lesions with breast cancer subtypes are less known. Kim et al. [8] reported that breast cancer subtypes consisted of 48.1% luminal (HER2-negative), 22.2% hormone receptor (HR)-negative and HER2-positive, 22.2% HR-positive and HER2-positive, and 7.4% triple-negative (ER/PR/HER2). These frequencies of breast cancer subtypes are similar to the subtypes of DCISs and microinvasive carcinomas in our data, which had higher HER2-positive rates (HR-positive and -negative) than invasive breast carcinomas, which are usually approximately 15% [12]. Additionally, there was a lower triple-negative rate, which is usually 10–15% of invasive breast carcinomas. These results suggest that malignant NML lesions may be mainly in situ-based carcinomas.

## Summary and future perspectives

There are both benign and malignant NML lesions, and it is important to distinguish between these benign lesions (fibrocystic changes, adenosis, and hyperplasia) and malignant lesions (mainly DCIS). Additionally, it appears that the importance of detecting NML lesions depends on whether they are associated with microcalcification (comedo necrosis). Microcalcifications include both benign and malignant lesions, but abundant hyperechoic foci in NML lesions seen as microcalcifications, such as linear, pleomorphic calcifications, on mammography are most likely HG carcinoma in situ or micro-minimal invasion with comedo necrosis (typically HER2-positive or TNBC). Other malignant NML lesions may be typically LG DCIS. Sagara Y et al. reported that the survival benefit of LG-DCIS is lower than that of intermediate- and HG-DCIS for performing breast surgery [14]. Moreover, LG-DCIS may not be a fatal disease. Although the results are insufficient, LORIS [15], LORETTA [16], and other clinical trials for the active surveillance of “low-risk DCIS” are ongoing. Thus, we need to carefully assess NML lesions to avoid overdiagnosis and overtreatment from a pathological perspective. Future studies should further assess the relationships between NML lesions on US and their malignant potential including breast cancer subtypes and prognoses.

## Data Availability

Data sharing not applicable to this article as no datasets were generated or analyzed during the current study.
